# Evidence for Natural Products as Alternative Wound-Healing Therapies

**DOI:** 10.3390/biom13030444

**Published:** 2023-02-27

**Authors:** Rachael L. Moses, Thomas A. K. Prescott, Eduard Mas-Claret, Robert Steadman, Ryan Moseley, Alastair J. Sloan

**Affiliations:** 1 Melbourne Dental School, Faculty of Medicine, Dentistry and Health Sciences, University of Melbourne, Parkville, VIC 3010, Australia; 2 Royal Botanic Gardens, Kew, Richmond, Surrey TW9 3AB, UK; 3 Welsh Kidney Research Unit, Division of Infection and Immunity, School of Medicine, Cardiff University, Cardiff CF14 4XN, UK; 4 School of Dentistry, Cardiff University, Cardiff CF14 4XY, UK

**Keywords:** chronic wounds, bioactive small molecules from natural sources, wound dressings, clinical trial, plant, ulcer, antimicrobial

## Abstract

Chronic, non-healing wounds represent a significant area of unmet medical need and are a growing problem for healthcare systems around the world. They affect the quality of life for patients and are an economic burden, being difficult and time consuming to treat. They are an escalating problem across the developed world due to the increasing incidence of diabetes and the higher prevalence of ageing populations. Effective treatment options are currently lacking, and in some cases chronic wounds can persist for years. Some traditional medicines are believed to contain bioactive small molecules that induce the healing of chronic wounds by reducing excessive inflammation, thereby allowing re-epithelisation to occur. Furthermore, many small molecules found in plants are known to have antibacterial properties and, although they lack the therapeutic selectivity of antibiotics, they are certainly capable of acting as topical antiseptics when applied to infected wounds. As these molecules act through mechanisms of action distinct from those of clinically used antibiotics, they are often active against antibiotic resistant bacteria. Although there are numerous studies highlighting the effects of naturally occurring small molecules in wound-healing assays in vitro, only evidence from well conducted clinical trials can allow these molecules or the remedies that contain them to progress to the clinic. With this in mind, we review wound-healing natural remedies that have entered clinical trials over a twenty-year period to the present. We examine the bioactive small molecules likely to be in involved and, where possible, their mechanisms of action.

## 1. Introduction

Chronic wounds which result from impaired dermal wound healing can be categorised as being venous leg ulcers, diabetic foot ulcers or pressure ulcers [1,2]. These non-healing chronic wounds can have a significant impact on the patient, affecting mobility and increasing morbidity [1,2]. There is a considerable cost to healthcare systems worldwide in dealing with chronic wounds, with incidence increasing in line with rising obesity and diabetes rates [1,3,4].

Wound healing consists of a cascade of overlapping phases; haemostasis, inflammation, proliferation/re-epithelialisation and remodelling, performed in a highly controlled manner to ensure successful wound repair [5]. There are numerous alterations to this cascade in chronic wounds, which can also include intrinsic cellular changes between ‘acute’ healthy dermal wounds and chronic wounds; these alterations can result in chronic wounds persisting for months and in some cases over a year [1,6,7]. The longevity and chronicity of these wounds has a significant impact on patients, through pain, lack of mobility and increased morbidity [1]. In addition, there is a significant economic burden to healthcare systems from the treatment of chronic wounds, amounting to approximately 3% of the UK’s healthcare expenditure [3,4].

**Figure 1:**
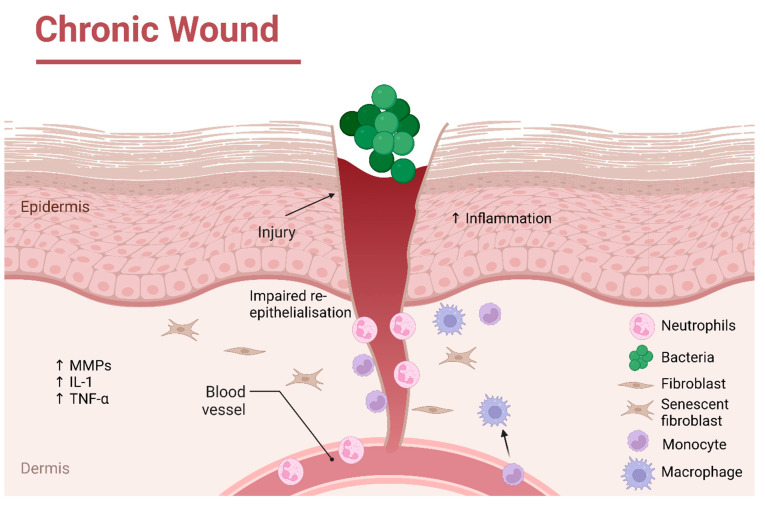
Schematic overview of the chronic wound environment, depicting key differences in the wound healing response compared to acute wounds. Created with BioRender.com.

One of the predominant features of chronic wounds is the prolonged inflammatory phase, where a detrimental feedback system recruit populations of neutrophils to the wound site, along with monocytes and pro-inflammatory M1 macrophages, thereby maintaining the persistent inflammatory state [8,9]. Compared to acute wounds, chronic wound exudate contains increased levels of matrix metalloproteinases (MMPs), which degrades vital growth factors required for extracellular matrix (ECM) deposition [8]. Additionally, there is an increased presence of pro-inflammatory cytokines present in chronic wounds, including IL-8, which further stimulates the chemotaxis of immune cells to the wound site, contributing to the detrimental feedback of inflammation [10]. This inflammatory environment impacts the functionality of the cells present at the wound site, with chronic wound fibroblasts reported to be senescent and displaying impaired wound-healing responses [11]. A key characteristic impairment of chronic wounds is the failure of the damaged epidermal barrier to undergo re-epithelialisation, with dysfunctional epidermal keratinocyte functions evident, including impaired migratory ability [6,12]. Impaired keratinocyte function has been associated with a reduced response to key growth factors, including epidermal growth factor (EGF), which is required for stimulating keratinocyte migration and subsequent re-epithelialisation. Conversely, EGF expression is increased in acute wounds, contributing to successful re-epithelialisation [13]. This absence of the protective barrier of the skin leaves the wound vulnerable to bacterial colonisation and subsequent infection, further impacting the chronicity of the wound, as depicted in the schematic image in Figure 1 [7,14]. Chronic wounds possess a high bacterial load, with the presence of biofilm at the wound site impacting the normal wound-healing response [14].

There is a wide array of treatment modalities available to tackle this clinical burden; however, they possess varying efficacy, with treatment options ranging from simple wound dressings to pharmaceutical growth factor therapies [11,15]. Chronic wound management includes the initial debridement of the necrotic tissue, before utilising one of the many treatment strategies. There are a variety of natural, semi-synthetic and synthetic wound dressings, which provide a scaffold for incoming cells, including collagen scaffolds, hyaluronan scaffolds and biosynthetic scaffolds. Some possess advantages over normal wound care strategies, such as the hyaluronan dressing, due to its role in promoting wound healing; however, others do not demonstrate any improvement over conventional wound care strategies, whilst also risking disrupting the wound bed through the repeated changing of dressings [15]. Others are also classed as bioactive or advanced wound dressings, as these are loaded with additional wound-modifying agents, such as antimicrobials and immunomodulatory moieties [16,17]. Negative pressure wound therapy removes wound exudate and contaminants through the use of suction, with this pressure drawing the wound edges closer together, aiding wound closure; however, this is a more costly treatment modality [11]. Another more costly option is the use of split-thickness autografts, involving donor site keratinocytes and expansion to form a graft. However, to avoid the risk of rejection, patient cells are used, resulting in another wound site for the patient [15]. Treatments can be applied in isolation or combination, such as negative pressure wound therapy used in combination with wound dressings [11].

Due to the complexity of chronic wounds, there are many limitations associated with current treatment modalities, including poor efficacy, incidence of wound recurrence or risk of tumour formation. Becaplermin gel, a growth factor therapy using a recombinant form of isoform PDGF-BB, aims to replace key degraded growth factors directly at the wound site; however, tumour formation has been reported following its use [15,18,19]. This highlights the importance of all the steps required to demonstrate the safety and efficacy of potential therapies. Current healthcare strategies are not adequately meeting this demand, due to the ever-increasing ageing populations and increased prevalence of obesity and diabetes. Therefore, new therapies with greater efficacy are required to ease the clinical burden, leading many researchers to direct their focus onto bioactive small molecules from natural sources, some of which have been used by indigenous populations for generations.

In remote communities, where modern healthcare access is limited, there are many reports on the use of plants, fungi or other natural materials, such as honey, which contain bioactive small molecules capable of promoting wound healing, with a number shown to possess potent bioactivity [20,21,22,23]. Reports by the World Health Organisation state that, in some countries, the use of bioactive small molecules from natural sources far outweighs the use of licenced pharmaceutical therapies for their healthcare needs; natural remedies have been used for a wide range of healthcare needs [20,21,22,23,24,25]. A key question that needs to be answered is: how effective are these natural remedies, and in each case, what are their bioactive constituents and what are their mechanisms of action? This review is divided into two sections; the first section focuses on clinical trials of bioactive small molecules from natural sources for wound healing within the last 20 years that contained more than 20 participants. The second section examines a selection of bioactive small molecules from natural sources with promising wound-healing potential, some of which are currently available as therapeutics, whilst others remain under development. 

## 2. Clinical Trials within Last 20 Years

A PubMed search, using the search criteria “ulcer” + “plant”, was performed to obtain details of the clinical trials carried out using bioactive small molecules from natural sources for wound healing in the last 20 years [26]. The phrase ulcer was used to select the most relevant articles due to the terminology used to describe different forms of chronic wounds, including diabetic foot ulcers, venous leg ulcers and pressure ulcers. From these search criteria, 75 articles were obtained; some were excluded due to their lack of relevance to this review, including oral or corneal wounds, along with those assessing preventative responses. Utilising the guidelines by the Food and Drug Administration (FDA) on what classifies as a Phase I study, clinical studies which met these baseline criteria, such as the inclusion of more than 20 participants, were included in this review assessing the potential of bioactive small molecules from natural sources for the treatment of chronic wound ulcers [27]. These selection criteria produced 16 clinical trials, which are summarised in Table S1. The most notable observation from these search criteria is the number of studies with a small participant sample size; some of the excluded studies were performed using participants undergoing wound care strategies through their healthcare provider, with anecdotal results obtained.

## 3. Diabetic Foot Ulcers

**Figure 2:**
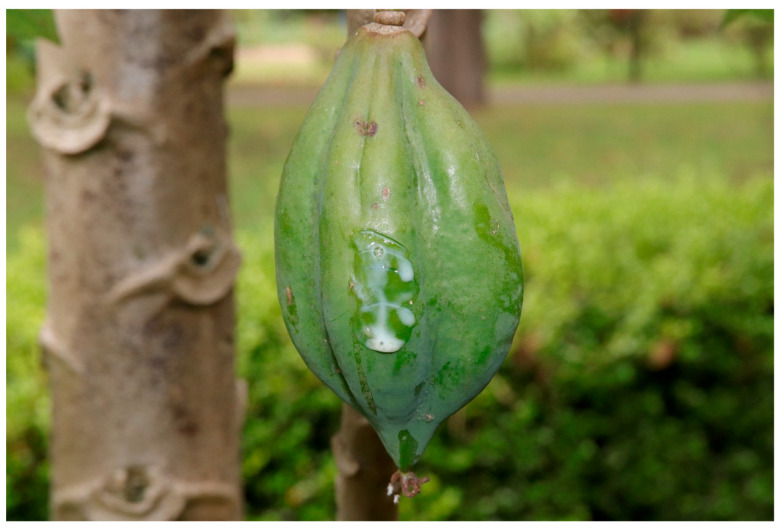
Photo of *Vasconcellea pubescens* A.DC (copyright held by Royal Botanical Gardens, Kew).

A study by Tonaco et al. (2018) assessed the potential of *Vasconcellea pubescens* A.DC., depicted in Figure 2 and referred to as *Vasconcellea cundinamarcensis* (ex-*Carica candamarcensis*) in this article and commonly referred to as Mountain Papaya, on diabetic foot ulcers [28]. This study included 50 participants in a randomised, double-blind trial, comparing the treatment outcome of the proteolytic fraction of *V. cundinamarcensis*, referred to as P1G10 in this study, to a control hydrogel. This clinical trial was a Phase II study included on the NIH clinical trials database, trial identifier: NCT03700580 [29]. The hydrogel control used as the comparison is currently used as a wound care treatment, providing a fair assessment of the novel therapy; no adverse side effects were reported following the application of P1G10. Any potential bias in reporting outcomes was prevented through randomisation, with patients randomly assigned to one of two treatment arms, in a blinded design; staff with no further role in the study were involved in preparing the treatment formulations [28,29]. *V. cundinamarcensis* was previously included in the genus *Carica*, but genetic information resulted in its reassignment to the *Vasconcellea* genus. It has also been shown to possess potential in burns, dermabrasion and incisional wounds, demonstrating wide applicability in wound repair treatments [30,31,32]. The efficacy of P1G10 is thought to be due to proteases in the plant, debriding necrotic tissue. Additionally, this proteolytic response may impair or disrupt the formation of biofilms, which affects the chronicity of the wounds [28]. Prior to the clinical study, this group performed animal studies assessing the impact of P1G10 on skin scald wounds on mice and determined the beneficial re-epithelialisation response was particular to P1G10 treatment, as another proteolytic enzyme, papain, did not demonstrate the same wound-healing efficacy [30]. In addition to the proteolytic response, P1G10 induced an early inflammatory response in a mouse cutaneous excision model, demonstrated by a significant increase in the presence of neutrophils within 3 days of treatment, a key response for clearing wound debris and bacteria [32]. In the clinical study, P1G10 was formulated within a Polawax dressing, used at 0.1% *w*/*w*; the vehicle delivery system was assessed for adverse responses prior to the study, but did not mention any potential beneficial responses from the vehicle alone. However, compared to the routinely used control hydrogel, 0.1% P1G10 demonstrated enhanced wound repair, with eleven and four patients for the P1G10 group, compared to five and three patients for the control group, respectively, exhibiting 100% or ≥80% wound closure overall. In addition to a greater proportion reaching 100% wound closure, the timeframe required was significantly reduced with P1G10 treatment, compared to controls [28,29].

A bark extract showing wound-healing potential is Pycnogenol^®^, which is the registered name for an extract from a French maritime pine, *Pinus pinaster* Aiton. Pycnogenol^®^ has been demonstrated to lower the blood glucose levels of patients with type 2 diabetes [33,34]. A clinical study was performed to evaluate the wound-healing response of Pycnogenol^®^ on diabetic ulcers. This study contained 30 participants split across three treatment groups and an untreated control [33]. The three treatment groups consisted of an oral capsule containing Pycnogenol^®^, a topical treatment involving the powder from the capsule applied to the ulcer, and a combination of both the oral and topical treatments. All four groups received the same level of ulcer care. Treatment with Pycnogenol^®^ significantly reduced the ulcer area within 6 weeks, across all three treatment groups. Interestingly, the combination treatment of oral and topical application induced a greater healing response, with 89% of ulcers successfully healed, compared to 61% healed in the untreated controls [33]. This corroborates the results seen in a smaller clinical study of Pycnogenol^®^ involving venous ulcers, with oral treatment and the combination of oral and topical application utilised in this study, with the combination treatment resulting in the greatest reduction in ulcer size within 6 weeks [35]. Rodent studies assessing the wound-healing response of Pycnogenol^®^ have shown enhanced healing, with a dose-dependent response on the healing time. 5% Pycnogenol^®^ induced wound healing within 12.1 days, compared to 15.3 days in the untreated control and 15.4 days in the carrier gel control [36]. Another diabetic rodent study compared the wound closure response following the topical application of Pycnogenol^®^ against an antiseptic cleanser (ethacridine lactate and silver sulfadiazine). Both treatments induced a greater reduction in wound size than the untreated control (44.58% reduction), with Pycnogenol^®^ stimulating a further reduction in wound size compared to the cleanser, 49.84% and 47.84%, respectively [37]. 

**Figure 3:**
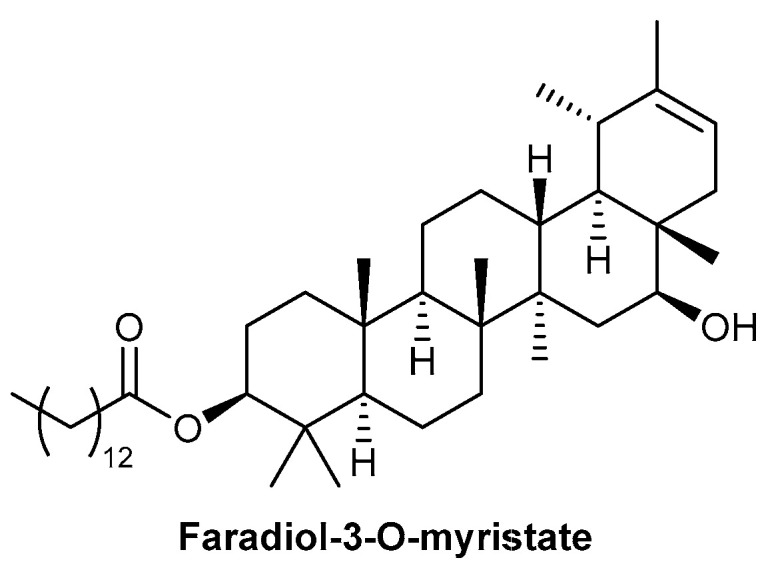
Chemical structure of faradiol-3-O-myristate, one of the major anti-inflammatory triterpenoid esters from *C. officinalis*.

A study was performed assessing the effect of *Calendula officinalis* L. hydroglycolic extract on diabetic foot ulcers. *C. officinalis* is commonly referred to as pot marigold, and is part of a genus of plants that has a long history of medicinal use, in particular as an anti-inflammatory treatment [38,39]. Faradiol-3-O-myristate is one of the major anti-inflammatory triterpenoid esters from *C. officinalis*, with the chemical structure depicted in Figure 3 [40]. As a prospective pilot study, all 41 patients enrolled into the study were treated with *C. officinalis* hydroglycolic extract; 78% of patients exhibited complete wound closure within 30 weeks of treatment, with a mean time of 15.5 weeks [38]. The presence of colonised bacteria within the ulcers was significantly decreased following treatment with *C. officinalis* hydroglycolic extract, with the reported pain experienced by the patients also reduced following treatment [38]. A larger, randomised study will be beneficial to follow up this pilot study, comparing it to standard wound care strategies and a vehicle-only control. This sentiment is also expressed in a systematic review on the effectiveness of *C. officinalis* for chronic wound treatment [41]. However, a significantly enhanced wound-healing response of venous leg ulcers was observed in a small clinical study, following treatment with an ointment containing *C. officinalis*, providing further reasoning to perform a larger, randomised clinical study [42]. An in vitro study aimed to determine the effect of *C. officinalis* on fibroblast proliferation and migration responses, with platelet-derived growth factor (PDGF) used as a positive control due to its role in wound healing, in particular, fibroblast chemotaxis to wound sites [43]. The use of a scratch wound assay demonstrated an increased presence of fibroblasts within the scratch site following treatment with *C. officinalis* extracts, comparable to the positive control, PDGF. The use of an antiproliferation agent, mitomycin C (5 µg/mL), produced equivalent responses, indicating that *C. officinalis* extracts induce wound repopulation responses through stimulating fibroblast migration [43]. 

A clinical study was performed to assess the efficacy of a polyherbal cream on diabetic foot ulcers compared to silver sulphadiazine cream, a standard wound care treatment. The polyherbal cream contained a variety of plant products (*Glycyrrhiza glabra* L., *Musa* × *paradisiaca* L., *Curcuma longa* L., *Pandanus odoratissimus* L.f., *Aloe vera* (L.) Burm.f. and *Cocos nucifera* L. oil), inducing anti-inflammatory, cell proliferative, wound contraction and antimicrobial properties [44]. This study was a non-randomised, non-blinded study, with 19 patients assigned to each treatment group; each treatment cream was applied following a wash, before applying a dressing [44]. Silver sulphadiazine cream was used as a comparison, as it possesses bactericidal activity. The polyherbal cream induced a similar wound-healing response, both in terms of ulcer size and healing duration, with 43.1 and 43.6 days to heal for polyherbal cream and the silver sulphadiazine cream, respectively [44]. The use of a comparison treatment was beneficial to evaluate the healing response, although no vehicle-only control was included, so it is not possible to distinguish between any responses the delivery vehicle may have exerted on the wound-healing outcomes. A larger randomised clinical study will provide greater information on the wound-healing benefits of using the polyherbal cream on diabetic foot ulcers. 

A randomised double-blinded clinical study by Najafian et al. (2019) assessed the response of a combination gel containing *Aloe vera* (L.) Burm.f. (*A. vera)* and *Plantago major* L. (Plantavera 10% gel) on diabetic foot ulcers. Due to both plants possessing beneficial wound-healing properties, the gel was comprised of 5% hydroalcoholic extract of *P. major* and 5% mucilage of *A. vera* [45]. A placebo control gel was included, with 20 patients randomly allocated to each group. The placebo gel contained the same base materials as the active treatment and was formulated to ensure the placebo gel was the same shape and colour as the Plantavera gel. Treatment with Plantavera gel induced a significant improvement in total ulcer score within 4 weeks, compared to the placebo control, demonstrated by 70% complete recovery in the treatment group and no complete recovery observed in the placebo group [45]. A rodent study assessing the effect of Plantavera gel on full-thickness skin wounds also demonstrated an enhanced wound closure response following treatment (7.67% closure/day), compared to the controls, which showed a similar rate of healing; placebo gel control (4.85% closure/day) and untreated control (5.65% closure/day). The treatment group induced full wound closure in most cases at a faster rate, compared to both controls [46]. The wound-healing effects of *A. vera* is discussed further in this review. *P. major* has been demonstrated through the use of an ex vivo porcine model to induce a dose-dependent wound-healing response, compared to a phosphate-buffered saline (PBS) control. The ethanol extract of *P. major* induced a greater enhanced response than the water extract in equivalent concentrations. Both extract preparations were dissolved in PBS to account for any vehicle effect [47]. A larger study assessing the effect of Plantavera gel over a longer time period is required to determine the wound-healing response of this combination gel, benefitting from two wound healing-enhancing plants. 

**Figure 4:**
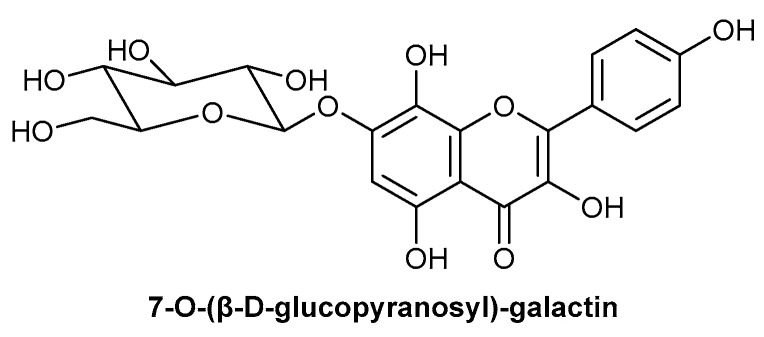
Chemical structure of flavonoid 7-O-(β-D-glucopyranosyl)-galactin, the active component of *A. pichinchensis* (Kunth).

A randomised double-blinded study by Romero-Cerecero assessed the effect of a 5% cream formulation of the native Mexican plant, *Ageratina pichinchensis* (Kunth), commonly referred to as Axihuitl, on diabetic foot ulcers [48]. The active component has been identified as flavonoid, 7-O-(β-D-glucopyranosyl)-galactin, with the chemical structure depicted in Figure 4 [48]. Treatment with *A. pichinchensis* demonstrated an enhanced wound-healing response, compared to a control treatment of 1% micronised silver sulfadiazine. However, this enhanced response was not determined to be statistically significant, potentially due to the low population size of 36 patients [48]. Additionally, a vehicle-only control was absent in the study, so it is not possible to distinguish between any beneficial impact of the delivery vehicle or the active component. A previous study by the same group assessed the effect of *A. pichinchensis* on venous leg ulcers; 34 patients were included in the study, with 17 in each of the treatment and control groups, and both were prepared in the same treatment group [49]. The reduction in ulcer size was significantly greater in the treatment group, compared to the control of 7% propylene glycol alginate. This difference in healing response between the two studies could be due to assessing venous ulcers, as opposed to diabetic foot ulcers, or the difference in control treatment included, with both controls used as a positive control for healing or antimicrobial responses [48,49]. In addition to the induction of the complete healing of venous ulcers with *A. pichinchensis* treatment over the study duration, the rate of healing was enhanced with *A. pichinchensis* treatment, shown by a 50% reduction in ulcer size by 1 month, compared to approximately 20% for the control [49]. A rodent diabetic model also demonstrated the enhanced healing response following *A. pichinchensis* treatment, compared to both a positive control wound-healing drug (5-methyl-1phenyl-2-(1H) pyridone) and negative control (vehicle only). This was shown by *A. pichinchensis* treatment inducing a 100% reduction in wound area within 11 days, compared to 70% and 40% for the positive and negative control, respectively [50]. 

## 4. Pressure Ulcers

**Figure 5:**
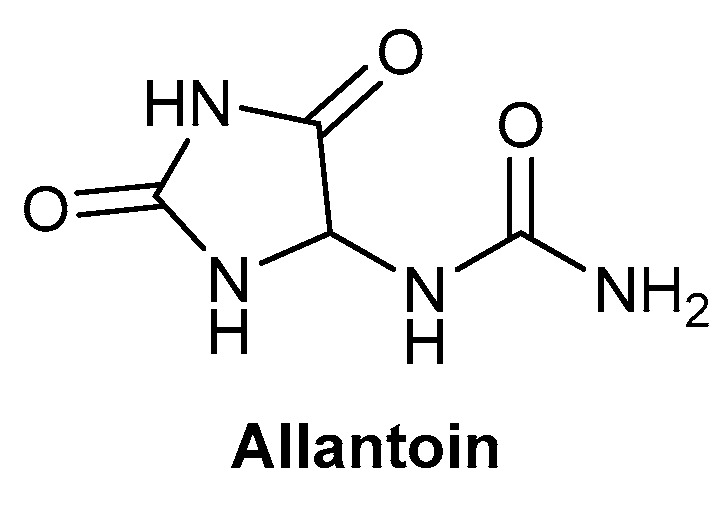
Chemical structure of allantoin, one of the major components responsible for the pharmacological effects of *Symphytum* × *uplandicum* Nyman.

A study by Stepan et al. (2013) assessed the potential of Symphytum Herba extract cream on pressure ulcers, decubitus bedsores, with bandages containing the cream changed every 2–3 days. All ulcers in this study were treated with Symphytum Herba extract cream, with no inclusion of an untreated control group [51]. Symphytum Herba extract cream is said to contain *Symphytum* × *uplandicum* Nyman, with allantoin reported as one of the major components responsible for the pharmacological effects, with the chemical structure depicted in Figure 5; it is typically referred to as Comfrey and is a perennial plant [51,52]. The study had a large patient cohort, consisting of 151 participants involved in the study until its completion, with 184 pressure ulcers present across the participants. At the completion of the study, 25–30 days, 170 ulcers were deemed as completely healed. It is noted that the wounds were disinfected at each bandage change, which could contribute to the efficacy of Symphytum Herba extract cream on the wounds. However, the high rate of successful wound repair shows great promise, along with the minimal incidence of skin sensitivity from application of Symphytum Herba extract cream, with only 1.2% of patients demonstrating local irritation [51]. 

A randomised clinical trial was performed assessing the use of a Norway spruce (*Picea abies* L.) salve applied in a sterile gauze on pressure ulcers, compared to a standard ulcer treatment of sodium carboxymethylcellulose hydrocolloid polymer with or without ionic silver (Aquacel^®^ or Aquacel Ag^®^), depending on the presence or absence of wound infection [53]. Despite 37 patients enrolling into the trial, only 22 remained for the full study duration, with a total of 29 ulcers across the patients; a higher proportion of patients and ulcers were in the salve group (13 and 18, respectively), compared to the control (9 and 11, respectively). The healing outcome of the salve group was almost double that seen in the control, with ulcers healed in 92% and 44% of cases, respectively. Additionally, the salve group typically healed in a shorter time frame, with complete healing evident in 94% of ulcers treated with the salve, compared to 36% in the control group [53]. These results were further corroborated in a clinical study assessing the salve in complicated, chronic surgical wounds, applied as a 10% (*w*/*w*) mixture of purified Norway spruce resin in a standardised salve base. Twenty-three patients were recruited to the trial, with all included in the salve treatment group, due to a lack of healing following surgery, providing no untreated control comparison [54]. All wounds healed following salve treatment, demonstrating the beneficial potential of *P. abies* salve; additionally, the cost of this treatment was calculated to be approximately 10–20% of that of sodium carboxymethylcellulose hydrocolloid polymer with or without ionic silver (Aquacel^®^ or Aquacel Ag^®^), indicating the economic benefit to healthcare providers [54]. In the previously described clinical trials, the presence of bacteria within the wounds for many patients prior to treatment with *P. abies* salve did not appear to affect the healing response. An in vitro study was performed to assess the response of the salve on *Staphylococcus aureus* [53,54,55]. Treatment with *P. abies* salve appeared to induce a bacteriostatic response on *S. aureus*, through a thickening of the bacterial cell wall, bacterial cell aggregation and reduced mitotic activity. Antimicrobial responses have been observed on a variety of gram-positive bacterial species, including antibiotic-resistant species, such as methicillin-resistant *S. aureus* [55,56,57]. 

## 5. Venous Leg Ulcers

A study by Romanelli et al. (2015) assessed the potential of *Triticum aestivum* L., referred to as *Triticum vulgare* in this article, on venous leg ulcers; this study assessed a variety of formulations of *T. vulgare*, commonly referred to as wheat plant, part of the *Graminaceae* family [58,59]. This study compared the effect of the delivery vehicle on wound response by comparing *T. vulgare* in one of five formulations: cream, impregnated gauzes, foam, hydrogel or dressing gel [58]. The participants were randomly assigned to a group, but there was an absence of a control group to compare the wound-healing efficacy against; this is acknowledged by the authors, who state the study was a pilot study focusing primarily on the medical device delivery system. Additionally, despite the study containing 50 participants, when split across the five medical devices, this results in only 10 participants per group. However, the authors also address this concern and find it sufficient for the pilot study, stating that it provides preliminary data to form the basis of a potential larger clinical trial. All five delivery systems/medical devices induced a reduction in wound ulcer size. However, there were variations across the groups; foam was the least effective at reducing ulcer surface volume, and dressing gel was the least effective at reducing total symptoms. The three best delivery systems were cream, gauze and hydrogel, all of which demonstrated an enhanced response [58]. Future studies with a larger patient cohort may determine if these differences are significant. Previous work by Romanelli’s group demonstrated the anti-inflammatory role of *T. vulgare* on inflammation marker matrix metalloproteinase-9 (MMP-9), which may benefit chronic wound resolution. However, this work focused on a microglial cell line, so it is not directly representative of dermal wound repair [59]. A study by Schiraldi’s group looked at the effect of *T. vulgare* extract on human keratinocytes (HaCaTs) and demonstrated a beneficial wound-healing response through the stimulation of keratinocyte proliferation and migration, indicating an enhanced re-epithelialisation response [60]. This enhanced migratory response was aligned with an early expression of MMP-2, compared to controls, allowing cell migration and wound repopulation to occur [60]. A clinical study by Saponati’s group demonstrated a greater reduction in lesion surface area and total symptom score, indicating an enhanced wound-healing outcome following treatment with *T. vulgare* gel (Fitostimoline) compared to a reference control of catalase-containing Citrizan gel, utilised in the treatment of burns, sores and ulcers [61]. This study used *T. vulgare* in the form of soaked gauzes and creams, both of which were shown to be more effective in the clinical trial performed by Romanelli et al. (2015) [58]. A double-blinded, randomised clinical study by Di Giulio et al. (2005) assessed the effect of Fitostimoline (*Triticum vulgaris*) on pressure ulcers and observed no significant difference in the re-epithelialisation of the wounds, compared to a placebo [62]. This was a large study consisting of 270 patients, where Fitostimoline treatment was applied in addition to recommended wound care treatment; the slight differences in outcomes were deemed not significant when adjusted to reflect risk of pressure ulcer development [62]. 

**Figure 6:**
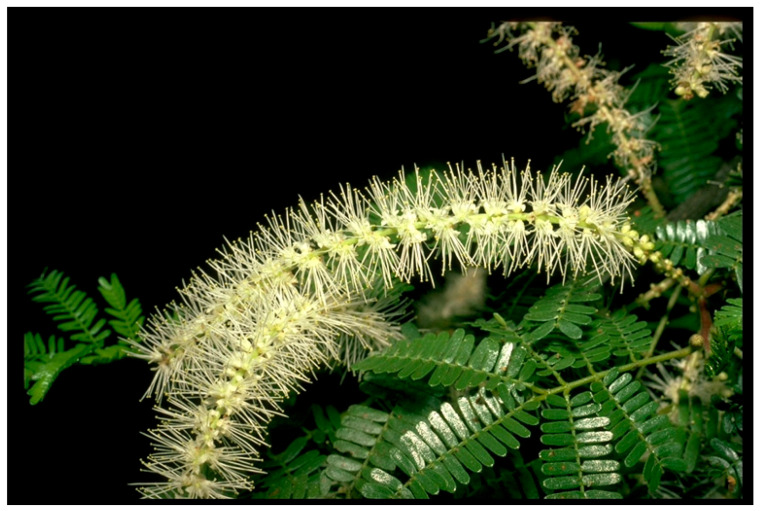
Photo of *Mimosa tenuiflora* (Willd.) Poiret (copyright held by Royal Botanical Gardens, Kew).

Clinical studies have been performed on *Mimosa tenuiflora* (Willd.) Poiret cortex extract, depicted in Figure 6 and obtained from the bark of the tepescohuite tree. Interest in this bark extract resulted from anecdotal and pre-clinical responses regarding its use on burn injuries and wounds [63,64]. These clinical trials assessed the response on venous leg ulcers, with both trials operating a double-blinded, randomised study design. The study by Rivera-Arce et al. (2007) demonstrated an enhanced healing response through a reduced ulcer area following treatment with the hydrogel containing the *M. tenuiflora* cortex extract, compared to the control hydrogel. Twenty patients were included in each of the treatment and control groups. Both the control and treatment groups used the same hydrogel formulation and treatment protocol. The control treatment resulted in an increased area of the venous ulcer, compared to the eventual closure of the ulcer with the bark extract treatment [63]. The study by Lammoglia-Ordiales et al. (2012) focused on the re-epithelialisation response of the *M. tenuiflora* cortex extract. The treatment protocol was the same as that used in the study by Rivera-Arce et al. (2007), with the hydrogel control included for comparison purposes [63,64]. Out of the 41 patients included in the study, only 32 were included in the analysis, due to lack of patient compliance or ulcer location complicating accurate measurement [64]. There was a slightly higher reduction in ulcer size with the *M. tenuiflora* cortex extract hydrogel, compared to the hydrogel alone, although this was not deemed significant. Histological analysis was performed on patients with a greater than 5 mm diameter wound remaining, although no significant differences were observed in terms of the presence of granulation tissue, fibrin or necrosis between the treatment and control groups. However, there was a significant increase in the re-epithelialisation in the treatment group, with 58% compared to the 39% seen in the control group [64]. The importance of establishing the epidermal layer is well-documented, due to its role as a protective barrier against microbial contamination [14]. One of the beneficial mechanisms of *M. tenuiflora* inducing an enhanced healing response is through antimicrobial activity. A review collating several in vitro studies demonstrated the antimicrobial activity of *M. tenuiflora*, shown through both bactericidal and bacteriostatic activities [65]. *M. tenuiflora* extracted through ethanol precipitation has been shown to stimulate proliferation of dermal fibroblasts by over 150%, compared to controls, indicating its beneficial use in wound healing [66]. 

A herbal ointment was assessed for its effect on venous leg ulcers; two different herbal treatments were included in the treatment group, with Plantoderm^®^ ointment containing alcohol extracts of *Calendula officinalis* L., *Symphytum officinale* L., *Achillea millefolium* L. and *Salvia officinalis* L. applied to the venous leg ulcers, and Fitoven^®^ gel (*Aesculus hippocastanum* L., *Melilotus officinalis* (L.) Lam., *Rosmarinus* L. and *Lavandula* L.) applied to the lower leg and any surrounding ulcers [67]. Wound care strategies were used in the control group through washing the wound site and using topical antibiotics when indicated. No vehicle-only control was included to determine any response from the delivery ointment. Following 7 weeks of treatment, the control showed a 35.65% reduction in ulcer surface, which was significantly less than the 42.68% ulcer surface reduction observed with the herbal treatments [67]. Additionally, the presence of bacterial species decreased in the herbal treatment group, compared to controls, with four patients in the treatment group showing no presence of bacterial isolates. In the treatment group across 17 patients, the presence of bacteria reduced from 25 to 16 isolates, compared to from 26 to 21 isolates in the control [67]. The plant extracts within the two herbal treatments have been reported to possess anti-inflammatory and antiseptic properties, along with inducing a significant decrease in ulcer surface measurement; a larger clinical study may provide further evidence on the benefit of using combination herbal therapies to stimulate wound repair of chronic wounds. 

**Figure 7:**
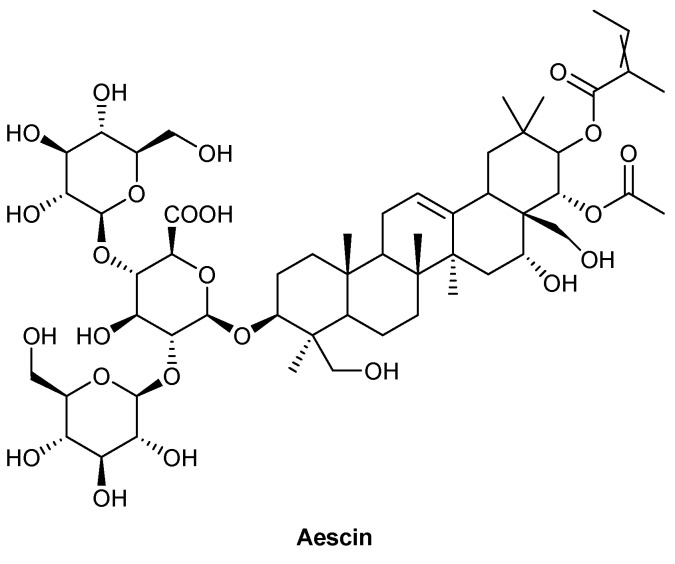
Chemical structure of aescin, the reported main active component of *A. hippocastanum* (L.), also referred as Horse chestnut seed extract.

Horse chestnut seed extract, *Aesculus hippocastanum* (L.), was administered as a tablet twice a day and assessed for its effect on venous leg ulcers, compared to standard wound care strategies, to determine any cost differences between the two treatments. Aescin, a mixture of triterpenoid saponins, was reported to be the main active component of Horse chestnut seed extract, with the chemical structure depicted in Figure 7 [68,69]. Patients were randomised into each treatment group and monitored over 12 weeks, with the placebo formulated using the same components as the active treatment and matched on colour and taste to avoid bias [68]. The main difference between the treatment groups was the decreased frequency of wound dressing changes required in the Horse chestnut seed extract treatment group, compared to conventional wound care treatment, with dressing frequency decreasing from an average of 2.1 per week at study initiation to 1.1 per week by the end of the study; compared to 2.41 and 2.48, respectively, for the standard wound care treatment [68]. This decrease has a resulting economic benefit, both with the reduced number of dressings used and the staff time needed to perform dressing changes. This study followed on from the clinical study by Leach et al. (2006), which found that despite a reduction in the number of dressing changes with Horse chestnut seed extract, thought to be due to a reduction in wound exudate production, there was no significant difference in ulcer size or healing outcome [69]. There were a variety of wound dressings used for both the treatment group and control group, which could have impacted the healing outcome. A larger, blinded study would be beneficial to determine if Horse chestnut seed extract induces a healing response. Additionally, using the same wound dressing across all patients would account for a variable potentially impacting on the wound-healing outcomes. A diabetic rodent study demonstrated altered MMP levels following treatment with an aqueous–ethanol extract of Horse chestnut. MMP-1 levels were shown to be significantly increased compared to controls, which was thought to aid keratinocyte migration during re-epithelialisation [70,71]. In contrast, treatment with Horse chestnut extract significantly decreased MMP-9 levels, thought to be beneficial due to the relationship between elevated levels, chronic inflammation and impaired wound healing in diabetic foot ulcers [70,72]. 

A non-randomised study was performed assessing the response of an ointment, Herbadermal^®^, on venous leg ulcers. Herbadermal^®^ is comprised of extracts of garlic (*Allium sativum* L.), St John’s wort (*Hypericum perforatum* L.) and calendula (*Calendula officinalis* L.), possessing a variety of beneficial properties, including anti-inflammatory and antimicrobial activities [73,74]. During this clinical trial, the epithelialisation was graded for the 25 patients present in the treatment group, with 15 patients in the control group [70]. There was a significant reduction in the size of the ulcers over the 7-week study duration, with a 99.1% improvement in epithelialisation following 7 weeks treatment with Herbadermal^®^. However, this study did not compare the treatment group with the control group, so no there was determination of whether this enhanced healing response is superior to the control [73]. 

A study by Panahi et al. (2015) assessed the potential of an *A. vera*/olive oil (*Olea europaea* L.) combination cream on a variety of chronic wounds, including venous ulcers, diabetic wounds and pressure ulcers [75]. As discussed later in this review, *A. vera* is part of the *Liliaceae* family of plants, typically observed in dry climates, that has been used in traditional medicine for centuries, with the Egyptians first utilising its medicinal properties for wound healing [75,76]. In the clinical study, participants were randomised into treatment groups, blinded to both participants and assessors. The control treatment included was phenytoin cream, a standard wound-healing treatment shown in a rodent study to accelerate wound repair response through upregulating PDGF expression and acting as a potent chemoattractant to dermal fibroblasts [75,77,78]. Both *A. vera* and olive oil treatments are known to play a role in wound repair, so their combination was likely to induce a beneficial response. The addition of olive oil to the combination cream is thought to prevent the dehydration of the skin through its emollient function [79]. Additionally, no notable adverse reactions were observed in this study, with 30 participants present in each treatment group [75]. Treatment with the *A. vera*/olive oil combination cream resulted in a significantly enhanced response over several wound healing criteria, including wound size, amount of exudate and peripheral tissue oedema. The phenytoin cream control induced a wound-healing response, but to a lesser degree than the treatment group; a vehicle-only control was not included in this study, so it was not possible to determine the response exerted from the delivery vehicle compared to the active treatment [75]. This enhanced wound repair response was corroborated by a further blinded randomised clinical study by Panahi et al. (2020), assessing its effect on the chronic inflammatory skin condition, atopic dermatitis [79]. Treatment with the *A. vera*/olive oil combination cream induced a greater reduction in the SCORAD severity index of patients with atopic dermatitis, compared to Betamethasone. Additionally, participants in the combination cream group reported a better quality of life, with all in the treatment group reporting an improvement, compared to less than half in the Betamethasone group [79]. 

The clinical studies discussed here demonstrate the potential of utilising bioactive small molecules from natural sources to stimulate wound repair in chronic wounds. The paucity of clinical studies within the last 20 years, found utilising the search criteria described previously, assessing the beneficial response of bioactive small molecules from natural sources for wound-healing outcomes, contrasts with the wide array used worldwide, particularly in remote communities with poor access to Western medicine [20,21,22,23]. A greater focus on those with potent bioactivity is vital to ease the clinical burden in the Western world, especially with an increased prevalence of antibiotic resistance, further compounding the chronicity of these impaired healing wounds [80]. The next section of this review will discuss some promising bioactive small molecules from natural sources at various stages of development as potential wound-healing treatments. 

## 6. Bioactive Small Molecules from Natural Sources with Promising Wound-Healing Potential

### 6.1. Manuka Honey

**Figure 8:**
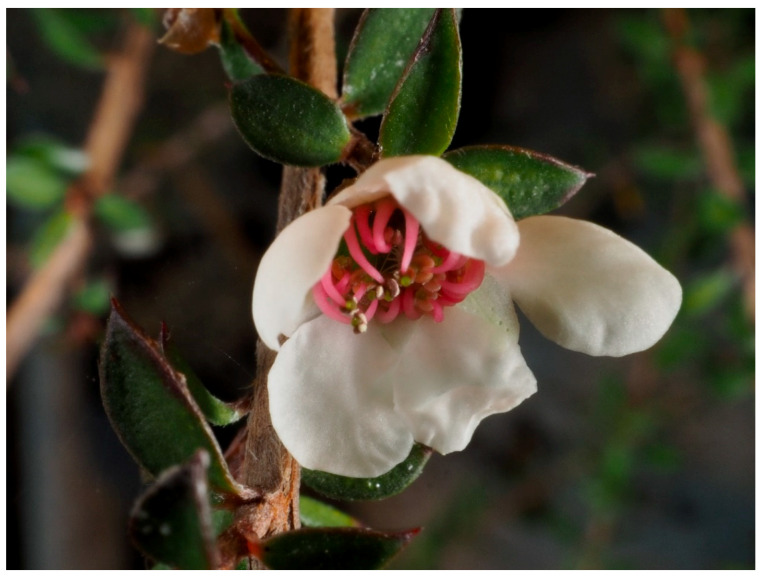
Photo of *Leptospermum scoparium* J.R.Forst. and G.Forst. (copyright held by Egon Krogsgaard and available under Creative commons licence: Attribution—NonCommercial CC BY NC).

Manuka honey was the first naturally derived product to be approved by the FDA for use in wound care, indicated for chronic wounds, burn injuries and surgical wounds [24,81]. Honey has been used in wound care for thousands of years, with records of its use in Ancient Egyptian times documented on the Edwin Smith papyrus between 2600 and 2000 BCE [82,83,84]. Manuka honey is a specific variety of honey generated by bees utilising the flower *Leptospermum scoparium* J.R.Forst. and G.Forst., depicted in Figure 8, native to New Zealand and Australia [24,85]. Honey has been demonstrated to possess numerous beneficial properties, including antibacterial and anti-inflammatory activities [85,86,87]. These responses are exerted through a variety of parameters, including osmolarity, peroxide activity, sugar content and presence of phytochemical compounds [85,88]. The high sugar content of honey exerts a bactericidal effect through dehydrating the bacterial cells, further aided by the high acidity of honey, which is outside the optimal pH range that bacteria typically grow in; these effects, along with the peroxide activity, are muted slightly at wound sites due to the presence of wound exudate [88]. 

**Figure 9:**
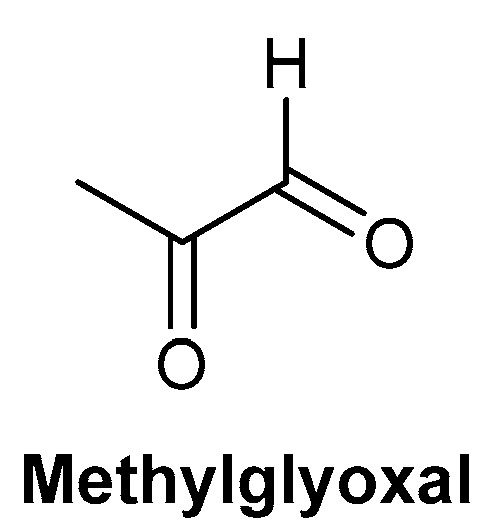
Chemical structure of methylglyoxal, the main antibacterial component of Manuka honey.

Manuka honey gained popularity as a therapy over other honey types due to its antibacterial activity, independent of its ability to release hydrogen peroxide; this ability to possess a non-peroxidase-dependent activity is beneficial, as it can withstand the necessary sterilisation process [85,89]. The active component of the non-peroxidase activity attributed to honey is methylglyoxal, with studies demonstrating the concentration of methylglyoxal is proportional to the antibacterial activity of Manuka honey. This corroborates studies that have determined methylglyoxal is the main active antibacterial component of Manuka honey; the chemical structure of methylglyoxal is depicted in Figure 9 [89,90,91]. Additionally, Manuka honey has been demonstrated to be active against antibiotic-resistant bacteria, including methicillin-resistant *Staphylococcus aureus* (MRSA), which burdens healthcare providers worldwide [92,93]. Despite the strong efficacy of Manuka honey on MRSA-infected wounds, honey is not a first-line therapy, typically only being used after exhausting other avenues. Further clinical studies on Manuka honey may remedy this, allowing clinicians to forgo antibiotics for topical honey therapy [94,95]. In comparison to antibiotics used in the treatment of wounds, Manuka honey does not appear to induce resistance in bacteria [92,93,94,95,96]. Additionally, it can target a wide range of both gram-positive and gram-negative bacteria, acting as an antimicrobial for strains typically observed in chronic wounds, including *S. aureus* and *Pseudomonas aeruginosa* [95,96,97].

The application of medical-grade honey, including Manuka honey, is typically through single-use wound dressings, due to their convenience and ability to remain at the wound site for a prolonged period [81,86]. There is a large variety of dressings with FDA approval for wound care, with variations in the proportion of Manuka honey present on the wound dressing, marketed as Medihoney™ [86]. A clinical trial assessing the response of Medihoney™ on chronic wounds observed successful wound closure in 90% of patients. Another study demonstrated a clinically significant reduction in healing time with Medihoney™, compared to standard wound-healing dressings, with 32 days and 46 days, respectively, to reach 50% reduction in wound area [98,99]. 

Some studies have advocated for a combination of honey wound dressings alongside antibiotics in instances where sepsis is a concern. Combination therapy was demonstrated to result in a synergistic response in scenarios where antibiotic resistance was previously observed [93,100]. The high concentration of Manuka honey within the variety of FDA-approved wound dressings has been shown to be sufficient to maintain an effective concentration at the wound site, even when diluted by wound exudate [100]. An additional benefit of utilising honey as a wound care therapy is its ability to debride wounds, negating the need for surgical debridement [101]. This process occurs through stimulating autolytic debridement, inducing an osmotic response, drawing water from the cells and subsequently increasing the hydration at the wound site; this hydration is required to soften the slough at the wound site, and along with the denaturation of fibrin, allows the slough to detach [101].

Numerous studies have been performed on the efficacy of Manuka honey on wound healing, comparing it against other standard therapies. A systematic review of these findings was compiled to assess the efficacy of honey-based therapies; however, some of these studies were shown to be of low quality due to low sample sizes or not accurately reflecting standard wound care practices [84]. However, a study performed in South Africa comparing honey treatment to IntraSite^TM^ Gel, a hydrogel dressing, found no significant differences in the healing time between the two groups. Given that the cost of honey therapy is approximately 4% of the cost of IntraSite^TM^ Gel, this indicates the potential cost-effectiveness of honey therapy over other commercially available products [102,103]. There are several clinical trials listed on the NIH government site assessing the response of Manuka honey on a variety of wound healing scenarios, including diabetic foot ulcers, pressure ulcers and burn injuries [104]. Only one of the trials listed includes study results (trial registration NCT02577900). This study compares three wound dressings, nanocrystalline silver alginate, Manuka honey alginate and a standard dressing, paraffin tulle, for the treatment of diabetic foot ulcers [104,105]. Both nanocrystalline silver alginate and Manuka honey alginate dressings had a greater reduction in the size of ulcer, compared to the standard treatment, with nanocrystalline silver alginate stimulating the greatest reduction [105]. This was a small study with 31 participants randomly allocated into each treatment group, resulting in 10/11 participants per group. However, it was of interest that there was a greater proportion of adverse events observed in the conventional treatment, compared to the intervention groups combined [105]. Another trial listed (trial registration NCT03391310) documented the results in a publication, demonstrating the reduced wound healing time with the treatment of Manuka honey dressing/gel compared to the standard treatment [104,106]. This study consisted of 99 participants, randomised into the honey or standard care group, with no adverse events recorded in the honey treatment group [106]. Both trials demonstrated the enhanced wound-healing capability of Manuka honey wound dressings compared to the conventional treatment, indicating their potential to be used as first-line wound care treatment. There is a lack of clinical trials comparing Manuka honey to other types of honey to determine if Manuka honey has superior wound-healing responses. However, several in vitro microbial studies have been performed assessing the bactericidal activity against a range of microbes [107,108]. One study demonstrated that Manuka honey was superior in its antibacterial action across a range of microbes, although the other locally produced and commercially available honeys demonstrated antibacterial activity to a similar degree as Manuka honey on some microbes [107]. A rodent study determining wound-healing responses was performed comparing lavender honey (*Lavandula x allardii*), lavender oil (*L. x allardii*) and canola oil to Manuka honey, using full-thickness excisional wounds. No significant difference in wound size was evident after 12 days across all treatment groups and the untreated control. However, Manuka honey- and lavender honey-treated wounds were shown to have a lower capillary volume, indicative of a more mature wound site [108]. There is a strong need for a clinical study comparing Manuka honey to the varieties included in the antibacterial assays and rodent studies, to determine whether Manuka honey is clinically superior to other honeys. 

### 6.2. Aloe vera

**Figure 10:**
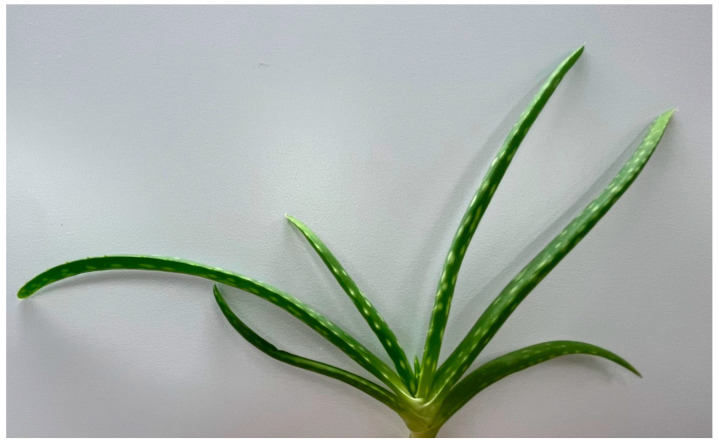
Photo of *Aloe vera* (L.) Burm.f. (copyright held by Royal Botanical Gardens, Kew).

**Figure 11:**
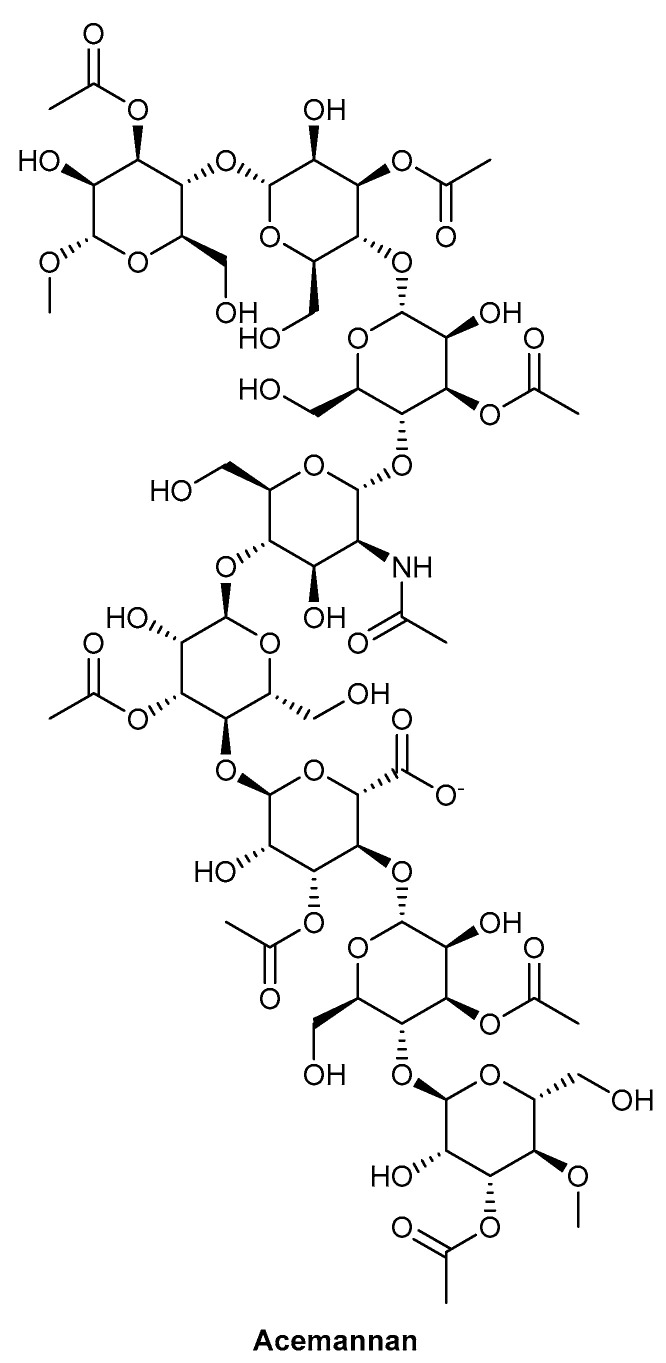
Chemical structure of acemannan, one of the key polysaccharides of *Aloe vera* (L.) Burm.f.

*Aloe vera* (L.) Burm.f., depicted in Figure 10, has been used for many years as a traditional medicine for a variety of therapies, including antimicrobial, anti-inflammatory, detoxification and digestion. In this review, we focus on the use of *A. vera* as a treatment for non-healing chronic wounds [109]. The *Aloe* species are typically found in dry climates, including Africa and India; the leaves present on *A. vera* contain a gel in the centre, which is the component primarily used in wound-healing therapies [109,110]. There are multiple active components within *A. vera* contributing to its wound-healing potential, including a number of polysaccharides. Acemannan is a polysaccharide reported to be one of the active components contributing to fibroblast proliferation and type I collagen synthesis. The chemical structure of acemannan is depicted in Figure 11 [110]. Care needs to be taken with the use of the skin of *A. vera* plants for medicinal uses, as it has been reported to possess mutagenic properties, thought to be due to the presence of anthraquinones within the skin. This was demonstrated through a study assessing whole-leaf extracts, with higher concentrations resulting in the formation of intestine carcinomas in rats [111,112]. A study by Fox et al. (2017) demonstrated that both the gel and whole-leaf material exerted a beneficial wound repopulation response. However, the *Aloe* gel treatments were superior to the whole-leaf materials in the induction of wound closure, assessed using an in vitro scratch wound assay with epidermal keratinocytes (HaCaTs) [113]. Due to the importance of establishing the protective outer barrier of the skin, stimulating the migration of epidermal keratinocytes over the wound site is essential for the re-epithelialisation process. This allows the re-established epidermis to prevent water loss from the tissue, whilst also providing a barrier to any microbial organisms [5,113]. 

Another important aspect of wound repair is the proliferation and migration of fibroblasts into the wound site and the subsequent deposition of extracellular matrix, replacing the temporary fibrin scaffold [5]. The effect of *A. vera* on fibroblast function was assessed by Shafaie et al. (2020) through use of the colorimetric MTT viability assay. The addition of *A. vera* gel was shown not to impair the viability of the fibroblasts and even appeared to stimulate their proliferation, particularly at 24 h. *A. vera* gel also appeared to stimulate the migration of fibroblasts into the denuded space within 24 h, assessed using an in vitro scratch wound assay; however, part of this enhanced migratory response could be a result of the induced proliferative potential [114]. However, the gene expression of integrins α1 and β1 significantly increased following treatment with *A. vera* gel, which corroborates the wound repopulation response observed in the scratch assay due to their role in fibroblast migration [114,115]. Therefore, *A. vera* gel could stimulate the proliferation and migration of fibroblasts independently, resulting in a further enhanced response. 

A randomised clinical study performed by Khorsani et al. (2009) assessed the potential of an *A. vera* cream against topical silver sulfadiazine on burn injuries. Silver sulfadiazine is used to prevent the microbial contamination of the burn site but impairs wound healing [116]. Given the long history associated with the use of *A. vera* on minor burns, there was validity in the assessments; a systematic review indicated a reduction in the healing time of burn injuries following *A. vera* treatment [117]. The clinical study by Khorsani et al. (2009) demonstrated this reduction in healing time following *A. vera* application when compared to silver sulfadiazine; all 30 patients (100%) in the *A. vera* group, compared to 80% of the silver sulfadiazine group, demonstrated complete wound healing with 19 days [116]. This healing difference was more pronounced at the healing rate within 16 days, with 83% and 23% for the *A. vera* group and silver sulfadiazine group, respectively [116]. *A. vera* has been included in combination treatments alongside other natural products possessing beneficial wound-healing properties, an example being the ointment containing honey (70%), *A. vera* (20%) and peppermint (*Mentha* × *piperita*; 10%). The use of this ointment, compared to a control of petroleum jelly, appeared to induce an enhanced wound-healing rate [118]. A rodent study assessing a hydrogel preparation of *A. vera*, Restauder^®^, demonstrated a significantly enhanced reduction in wound size through 91.6% wound reduction, compared to 83.99% reduction in the untreated control [119]. A benefit of this hydrogel preparation is being able to exert this healing response using previously manufactured batches, as opposed to freshly obtained *A. vera*, increasing the availability of *A. vera* products within the community. 

An in vitro study assessing the effect of *A. vera* on dermal fibroblasts and epidermal keratinocytes observed a significant stimulation of fibroblast and keratinocyte proliferation following treatment with 3% or 1% *A. vera*, respectively [120]. These same concentrations of *A. vera* also significantly stimulated the migration of fibroblasts and keratinocytes into denuded scratch sites within 24 h, compared to controls [120]. As discussed previously, the migration of keratinocytes is vital for re-epithelialisation, along with proliferation at the wound site. Additionally, the migration of fibroblasts to the wound site and subsequent proliferation is required to synthesise the extracellular matrix needed to replace the temporary fibrin scaffold produced following injury [5,121]. 

### 6.3. Tree Latex, Plant Exudates and Bark Extracts

In addition to the tree barks previously discussed, *Caesalpinia ferrea* Mart bark, located in north and northeast Brazil, has demonstrated accelerated healing in excisional wounds on Wistar rats [122]. This response was exerted through a reduction in inflammatory proteins, with a reduced infiltration of inflammatory cells observed through histological studies. Inflammation is an essential process in wound healing, but excessive or prolonged inflammation can induce a negative feedback system of ECM degradation, preventing successful wound resolution [8,122,123]. In addition to tree barks possessing beneficial wound-healing properties, tree saps have also been shown to accelerate wound healing.

The ethnopharmacological approach to discovering novel wound-healing therapies from nature is exemplified by research carried out at the Royal Botanic Gardens, Kew. Researchers there have been working with indigenous communities in Papua New Guinea to investigate the potential of traditional plant medicines used to treat cutaneous ulcers [124,125,126]. These skin ulcers are infected with a variety of bacterial species and are extremely common in children in Papua New Guinea, normally presenting as circular ulcers on the lower leg [126]. Although the pathogenesis of these ulcers is not yet fully determined, *Haemophilus ducreyi* and *Streptococcus pyogenes* are believed to be important bacterial pathogens. Children living in rainforest villages typically walk barefoot through rainforest mud, and small scratches on their ankles or lower leg quickly become infected. The early treatment of these ulcers using a topical antiseptic cream would be highly effective if it were not for the fact that such medicines are not readily available in remote rainforest areas. The aim here is not to identify a lead molecule from a plant but instead, through carrying out clinical trials, to provide an evidence base sufficient to justify the topical application of wound-healing plant saps as first-line treatments. They key advantage that plant medicines provide in this scenario is that they are found growing in villages and can be used to treat early-stage infections. By contrast, conventional treatments, such as antibiotics, must be sought in community clinics, which in most cases require walking many miles barefoot to reach. With this in mind, several plant saps have been investigated in vitro and one species has been tested in a clinical trial. 

The exudate of *Lepiniopsis ternatensis* Valeton was assessed in vitro [124]. Several wound-healing assays were performed, which demonstrated a stimulation of fibroblast proliferation, a beneficial response due to the impaired proliferative ability of chronic wound-derived fibroblasts [124,127]. Additionally, TNF-α secretion from granulocyte-macrophage colony-stimulating factor (GM-CSF)-stimulated M1 macrophages was inhibited following treatment with the highest concentration (1% *v*/*v*) of *L. ternatensis* sap [124]. Increased TNF-α is associated with elevated MMP expression, a hallmark of chronic wounds, driving ECM degradation; this inhibition in TNF-α may play a part in reducing the chronicity of the ulcers [128]. 

In another study of a Papua New Guinea plant medicine, the exudate of *Cypholophus macrocephalus* (Blume) Wedd was evaluated, again due to its documented traditional use as a topical treatment for cutaneous ulcers by the indigenous population [125]. Human-derived neutrophils and macrophages treated with this exudate demonstrated increased IL-6 release, equivalent to stimulation with lipopolysaccharide (LPS) and GM-CSF, respectively. There was a dose-dependent stimulation of TNF-α release, observed in both neutrophils and macrophages [125]. 

**Figure 12:**
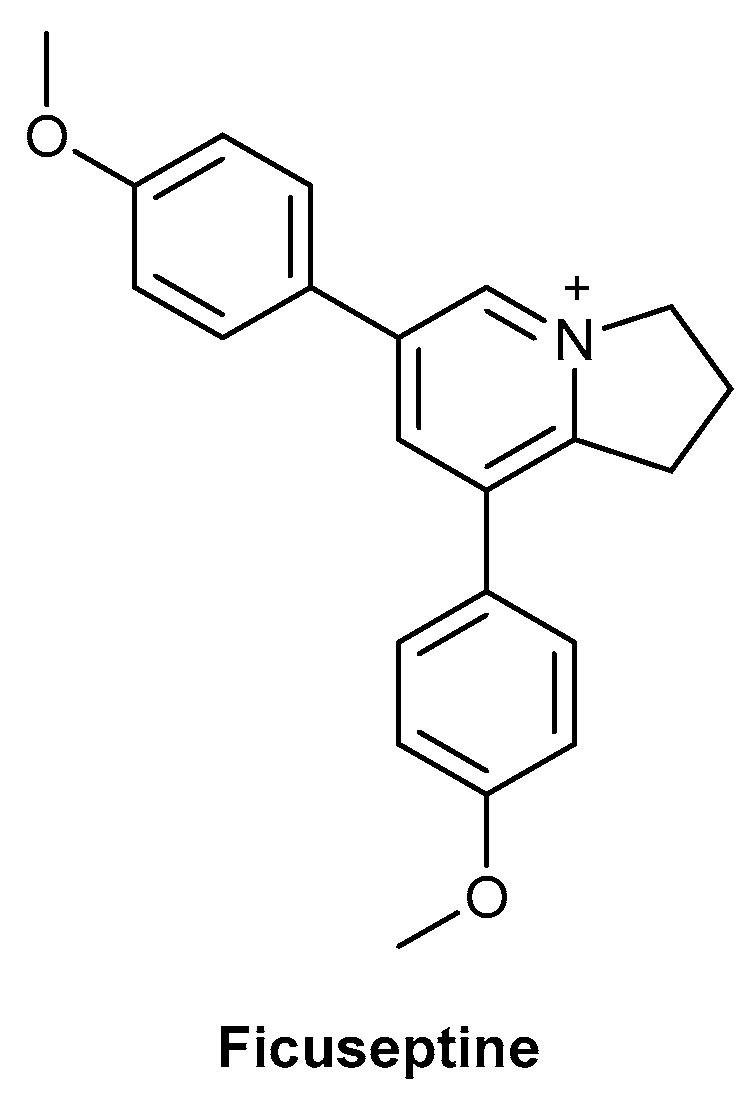
Chemical structure of ficuseptine, an alkaloid present in *Ficus septica* Burm.f.

In contrast to the plant exudates described above, the exudate of *Ficus septica* Burm.f. which contains the alkaloid ficuseptine (Figure 12) showed both antibacterial and anti-inflammatory effects in vitro. A significant dose-dependent decrease in the expression of IL-6 and TNF-α, from neutrophils and M1 macrophages, was observed. The latex of *F. septica* is used by the Kaulong-speaking population in Papua New Guinea as a topical treatment for infected cutaneous ulcers. Interestingly, a significant increase in IL-6 was observed at the lowest concentration, indicating the possibility that this exudate can exert a pro- or anti-inflammatory response, depending on the concentration used [126]. A clinical trial was carried out to assess the efficacy of *F. septica* exudate as a topical treatment compared with Savlon antiseptic cream or simply washing the skin ulcers with soap and water. The cluster randomised trial comprised 150 patients, with 50 patients per treatment arm. Ulcer images taken at baseline and days 7 and 14 were blinded and then assessed by three dermatologists. At day 14, the *Ficus* exudate was found to be non-inferior to both Savlon cream and soap and water treatment, in terms of its efficacy for healing/improving the ulcers.

In Northern Africa and the Middle East, date palm sap (*Phoenix dactylifera* L.) has been used for thousands of years as a natural remedy for anaemia, respiratory infections, oedema and wound healing [129,130]. A study utilising sap from the Beser variety of *Phoenix dactylifera* L., sourced from Tunisia, demonstrated accelerated wound healing in Wistar rats with experimentally induced wounds. Histological analysis showed regeneration of the tissue with the presence of fibroblasts, blood vessels and collagen fibres, compared to an absence of tissue regeneration in the untreated control [129]. Additionally, *P. dactylifera* possesses a high total antioxidant capacity and polyphenol content, contributing to an antioxidant and anti-inflammatory response. Different varieties of *P. dactylifera* possess different proportions of polyphenols, resulting in differing cellular responses [129,130,131,132]. 

### 6.4. Epoxy-Tiglianes

**Figure 13:**
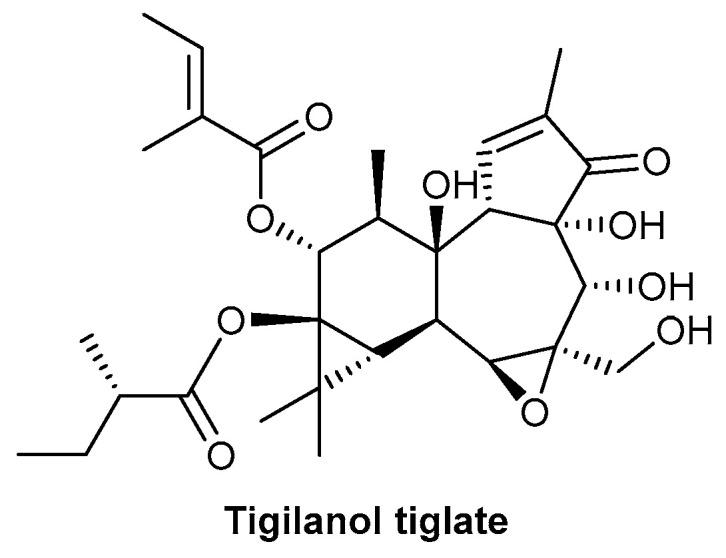
Chemical structure of tigilanol tiglate, the main component of *Fontainea picrosperma*.

Epoxy-tiglianes are a novel class of diterpene esters discovered and isolated from the Australian rainforest plant *Fontainea picrosperma* by QBiotics Group [133,134]. The prototype epoxy-tigliane, EBC-46 (now known as tigilanol tiglate), was initially studied for its potential as an anticancer therapy for application in treatment of both humans and companion animals; the chemical structure of tigilanol tiglate is depicted in Figure 13. The drug is currently being evaluated in Phase II human clinical trials as a local treatment for a range of cancers and has recently been approved as a veterinary pharmaceutical in the USA, Europe, the United Kingdom and Australia for treating non-metastatic, canine mast cell tumours (marketed under the tradename STELFONTA^®^) [133,134,135,136]. EBC-46 is a potent cellular signalling molecule with the activation of PKC, in part, responsible for its efficacy [137,138]. 

In addition to ablation of the treated tumours, veterinary and human studies have demonstrated that the drug induced an exceptional wound-healing response at the treatment site following tumour destruction; this was further corroborated with in vitro evaluation of epoxy-tiglianes on epidermal keratinocyte wound-healing functions [137,139]. These studies demonstrated significant proliferative and wound repopulation abilities, shown through MTT colorimetric assay, cell cycle analysis by Flow Cytometry and in vitro scratch assays, respectively; this enhanced wound-closure response resulted from the independent stimulation of proliferative and migratory activities, determined through use of antiproliferative agent, mitomycin C [139,140]. Global gene analysis on epidermal keratinocytes treated with EBC-46 showed the induction of several genes involved in driving wound repopulation through inducing the gene expression responsible for cell cycle progression, proliferation and migration. These differentially expressed genes corroborate the rapid re-epithelialisation response evident in veterinary case studies [137,139]. Matrix metalloproteinases (MMPs) also play a key role in wound repair through facilitating the migration of epidermal keratinocytes over the underlying dermal matrix during re-epithelialisation [71]. Following EBC-46 treatment, MMP-1, MMP-7 and MMP-10 activities were significantly increased; this corroborates the enhanced keratinocyte migration and wound repopulation responses observed during the scratch assays and the observations from the human and veterinary clinical studies [137,139]. These epoxy-tigliane-induced effects on keratinocytes in vitro have been shown to be the result of PKC activation, in particular, the classical PKC isoforms. 

Given the promising responses observed with these novel epoxy-tiglianes, both in vitro and in the clinic, there is future potential for their use to replace or complement existing wound-healing therapies, especially in difficult-to-treat chronic wounds. Further studies are being conducted to determine the precise mechanism of action of these novel pharmaceuticals to progress as a viable pharmaceutical therapy. 

## 7. Conclusions

Within the last 20 years, only 16 clinical trials were obtained, using the PubMed search described above, on the use of bioactive small molecules from natural sources for chronic wound treatment for diabetic foot ulcers, pressure ulcers and venous leg ulcers [26]. The limitation of this PubMed search could be the use of the keywords “plant” and “ulcer” to obtain the clinical trials detailed in this review; a few variations in these search criteria were assessed, with this combination producing the greatest number of relevant articles. The choice of relevant articles was based on the FDA guidelines for Phase I studies to determine the inclusion/exclusion of trials to be reviewed; this provided baseline criteria for inclusion, including the number of participants involved in the clinical studies. There was a paucity of studies obtained using the NIH clinical trials database to search for studies involving the plants described in this review article. Additionally, of those that were present, few were completed studies and had posted results. This contrasts with the huge number of natural therapies used by indigenous communities across the world, with knowledge of the active components of local flora often passed down through the generations. 

Bioactive small molecules from natural sources offer a variety of benefits, both in remote communities and within modern healthcare systems, such as ease of access in remote communities and potent bioactivities, such as anti-inflammatory and antimicrobial actions. Additionally, as discussed in some of the studies included in this review, there is the potential for combination therapies, both a combination of bioactive small molecules from natural sources and combination with other treatment modalities, such as antibiotics. The use of these combinations could result in a synergistic response, resulting in an enhanced wound-repair response, both benefitting the patient and easing the clinical burden on the healthcare system. Scoping natural compound-based folklore medicines for the screening, isolation and characterisation of active compounds and their subsequent translational development as novel chronic wound therapies will greatly benefit healthcare systems around the world due to the ever-increasing prevalence of chronic wounds.

There are multiple challenges involved in the development of novel therapeutics, including safety and efficacy assessment, along with the determination of the optimal dosage range. Additionally, the production of therapeutics is an expensive process, with production costs potentially varying depending on the source of bioactive small molecules. As these bioactive small molecules are from natural sources, there is the additional challenge of either sourcing or cultivating a sufficient quantity of the natural source of interest or the synthesis of the key bioactive molecule. A benefit of using bioactive small molecules from natural sources which have been previously used by indigenous populations is the knowledge about observed toxicity or adverse effects following their use and providing an indication of their wound-healing responses prior to in vitro assessment and subsequent clinical trials.

There are many in vitro and rodent in vivo studies on bioactive small molecules from natural sources, but there appears to be a gap in their progression to human clinical trials. Whilst these studies are of great interest and necessity, to progress as a medicinal therapy, clinical trials are required to confirm the efficacy of novel therapies. As discussed extensively in this review, there are a wide variety of bioactive small molecules from natural sources available which possess beneficial bioactivities and are currently used across the world; greater uptake of these natural therapies could help address the rising clinical burden associated with chronic wound management.

## Data Availability

Not applicable.

## Supplementary Materials

The following supporting information can be downloaded at: https://www.mdpi.com/article/10.3390/biom13030444/s1, Table S1: Summary of clinical trials carried out using natural products for wound healing in the last 20 years sourced using a PubMed search, using the search criteria “ulcer” + “plant”.
