# Supplementary material for: Evidence for Natural Products as Alternative Wound-Healing Therapies
Source: Biomolecules. 2023 Feb 27;13(3):444. doi: 10.3390/biom13030444 (PMC10046143; doi:10.3390/biom13030444)
Supplement: Supplementary file 1 [file biomolecules-13-00444-s001.zip › biomolecules-2039269-supplementary.pdf]

Table S1: Summary of clinical trials carried out using natural products for wound healing in the last 20 years sourced using a PubMed search, using the search criteria "ulcer" + "plant".

Test substance	Number of participants	Randomised /blinded	Outcomes	Reference
**Diabetic Foot Ulcers**				
Vasconcellea pubescens A.DC. (*Vasconcellea cundinamarcensis* (ex-*Carica candamarcensis*)) (Mountain Papaya)	50	Randomised, double blinded	Phase 2 clinical study. Demonstrated enhanced wound closure ability compared to control (currently used wound care treatment), with 11 patients vs 5 exhibiting 100% wound closure, respectively.	Tonaco et al. (2018)
Pinus pinaster Aiton (Pycnogenol®) (French maritime pine)	30	Randomised	Combination of oral and topical treatment resulted in 89% healed compared to 61% healed in untreated control.	Belcaro et al. (2006)
Calendula officinalis L. hydroglycolic extract (pot marigold)	41	All treated with test compound	78% of patients exhibited complete wound closure within 30 weeks of treatment.	Buzzi et al. (2016)
Polyherbal cream containing (*Glycyrrhiza glabra* L., *Musa × paradisiaca* L., *Curcuma longa* L., *Pandanus odoratissimus* L.f., *Aloe vera* (L.) Burm.f. and *Cocos nucifera* L. oil)	38	Not randomised	Polyherbal cream induced a similar wound healing response compared to the silver sulphadiazine cream, for ulcer size and healing duration, with 43.1 and 43.6 days to heal for each, respectively.	Viswanathan et al. (2011)
Combination gel containing *Aloe vera* (L.) Burm.f. (*A. vera)* and *Plantago major* L. (Plantavera 10% gel)	40	Randomised, double blinded	Plantavera gel induced a significant improvement in total ulcer score compared to placebo control within 4 weeks, with 70% complete recovery compared to no complete recovery, respectively.	Najafian et al. (2019)

Ageratina pichinchensis (Kunth) (axihuitl)	34	Randomised, double blinded	Reduced time needed for complete wound healing with *A. pichinchensis* extract (65.47 ± 47.08 days) compared to control (77.46 ± 50.8 days).	Romero-Cerecero et al. (2015)
**Pressure Ulcers**				
Symphytum × *uplandicum* Nyman	151 participants, 184 pressure ulcers	All treated with test compound	At the completion of the study (25-30 days), 170 out of a total 184 pressure ulcers were deemed as completely healed.	Stepan et al. (2013)
Picea abies L. (Norway spruce)	22 participants, 29 pressure ulcers	Randomised	Complete healing evident in 94% of ulcers treated with the *P. abies* salve, compared to 36% in the control group.	Sipponen et al. (2008)
**Venous Leg Ulcers**				
Triticum aestivum L., (*Triticum vulgare)* (Wheat plant)	50	Randomised into delivery vehicles; all treated with test compound	All five delivery systems/medical devices induced a reduction in wound ulcer size; the three best delivery systems were cream, gauze and hydrogel.	Romanelli et al. (2015)
Mimosa tenuiflora (Willd.) Poiret (*Leguminosae*) cortex extract	40	Randomised, double blinded	*M. tenuiflora* cortex extract demonstrated an enhanced healing response through reduced ulcer area following treatment with the hydrogel containing the control hydrogel.	Rivera-Arce et al. (2007)
Plantoderm® ointment containing alcohol extracts of (*Calendula officinalis* L., *Symphytum officinale* L., *Achillea millefolium* L., and *Salvia officinalis* L.)	32	Randomised	Plantoderm® ointment treatment showed 42.68% ulcer surface reduction, compared to 35.65% reduction with the control.	Binić et al. (2010)

Aesculus hippocastanum (L.) (Horsechestnut seed extract)	54	Randomised, triple-blinded	Horsechestnut seed extract treatment resulted in reduced wound dressing changes (1.1) compared to conventional wound care treatment (2.48) by the end of the study.	Leach et al. (2006)
Herbadermal® comprised of extracts of garlic (*Allium sativum* L.), St John's wort (*Hypericum perforatum* L.), and calendula (*Calendula officinalis* L.)	25	All treated with test compound	Herbadermal® treatment resulted in significant reduction in ulcer size over the 7-week study duration, with 99.1% improvement in epithelialisation.	Kundaković et al. (2012)
A. vera/olive oil (*Olea europaea* L.) combination cream	60	Randomised, double blinded	Treatment with the *A. vera*/olive oil combination cream resulted in a significantly enhanced response over several wound healing criteria, including wound size, amount of exudate and peripheral tissue oedema.	Panahi et al. (2015)
